# A comparative study on knowledge towards COVID-19 prevention among undergraduate students in Macao and Zhuhai, China

**DOI:** 10.7717/peerj.11833

**Published:** 2021-08-03

**Authors:** Xiaoyu Tao, Dong Chen, Rining Liang, Xiaoyu Zhang, Xi Yu, Sookja Chung, Yaqin Yu, Ying Xiao

**Affiliations:** 1 Faculty of Medicine, Macau University of Science and Technology, Macao, China; 2 Students’ Affairs Division, Zhuhai College of Science and Technology, Zhuhai, Guangdong, China; 3 Faculty of Health Sciences, Zhuhai College of Science and Technology, Zhuhai, Guangdong, China

**Keywords:** Knowledge, COVID-19, Undergraduate student, Macao, Zhuhai

## Abstract

In order to develop the strategy more suitable campus-based coronavirus disease 2019 (COVID-19) prevention programs for undergraduate students, it is critical to identify discrepancies in knowledge of COVID-19 prevention among students from different campuses in China. The present study examined the difference in preventive knowledge about COVID-19 pandemic in undergraduate students from two cities of Guangdong-Hong Kong-Macao Greater Bay Area (GBA), Macao Special Administrative Region (SAR), which had very few cases of COVID-19 patients, and Zhuhai, which is borders Macao SAR. In August 2020, two cohorts of undergraduate students from universities in Macao (*n* = 977) and Zhuhai (*n* = 2,818) were recruited for online. The self-rating questionnaire was used to gain information about their knowledge in COVID-19 prevention. Macao and Zhuhai students had similar correct rates in terms of heat inactivation conditions of SARS-CoV-2, (76.8% *vs.* 76.9%, *P* = 0.950), etiquette when coughing and sneezing (75.9% *vs.* 75.0%, *P* = 0.562), and use of disposable masks (92.2% *vs.* 90.6%, *P* = 0.126). However, students from Macao had significantly higher rates in correct use of disinfectants against SARS-CoV-2 (24.6% *vs.* 17.5%, *P* < 0.001) and in the route of transmission of SARS-CoV-2 (84.5% *vs.* 79.6%, *P* < 0.001) than those from Zhuhai. In conclusion, the knowledge level of COVID-19 prevention differs among undergraduate students from Macao and Zhuhai, which warrants an appropriate region-specific health education strategie for COVID-19 prevention.

## Introduction

The Coronavirus Disease 2019 (COVID-19) pandemic is still posing a global health threat with a sudden spurt of cases in some regions, such as India, U.S.A and UK. In China, there have recently been some small-size outbreaks in its northern parts, such as Shijiazhuang municipality and Wangkui county, although the pandemic has been well controlled in China since May, 2020. At present, COVID-19 vaccines are only allowed for emergency use in high-risk populations, such as the staff of the departments of Disease Control and Prevention Centers and front-line healthcare professionals in China. Therefore, precautionary measures are still the most effective way to prevent the viral infection of COVID-19 among the general population (Chinese Center for Disease Control & Prevention, 2020). As recommended by the World Health Organization (WHO), effective preventive measures include social distancing, quarantining, hand washing, and wearing disposable medical masks (World Health Organization, 2020).

In China, universities have resumed Zoom, face-to-face and/or hybrid classes after the successful containment of COVID-19 in fall of 2020 (Ministry of Education of the People’s Republic of China, 2020). However, it should be noted that the risk of COVID-19 cluster outbreak is still potentially high, since universities have large number of students in confound areas, such as small classrooms, shared rooms in dormitories and common facilities (Zhai et al., 2020). Therefore, it is of particularly importance to have students maintain correct precautionary behaviors during the outbreak and post-outbreak periods. To this end, it is necessary to assess students’ knowledge of COVID-19 prevention, since there is evidence that good knowledge of COVID-19 preventive measures is associated with appropriate practices towards COVID-19 outbreak (Zhong et al., 2020).

The Macao Special Administrative Region (SAR) and Zhuhai are two major bordering municipalities in Guangdong-Hong Kong-Macao Greater Bay Area (GBA) with distinct cultural, socio-economic, and educational backgrounds, as well as COVID-19 control measures. Universities in Macao also have resumed classes since fall of 2020 (Education & Youth Development Bureau, 2020), so universities of both municipalities face similar challenges of COVID-19 prevention. The differences in the knowledge level of COVID-19 prevention in undergraduate students between two municipalities and identification of underlying reasons for such differences may facilitate the development of more suitable campus-based COVID-19 prevention programs. Therefore, the present study compared Macao and Zhuhai undergraduate students’ knowledge of COVID-19 prevention measures.

## Methods

### Participants and procedures

This cross-sectional survey was conducted in August, 2020 before the commencement of the fall semester. Recruitment of participants and data collection were conducted online, because the field survey was not feasible due to online courses for university students of the two municipalities. The survey questionnaire was distributed *via* Wenjuanxing platform.

Several undergraduate students in Zhuhai and Macao were selected as primary seeds for this survey and participated in making local network to invite other undergraduate peers. The sampling approach is snowballing sampling. International students were not involved in the survey process.

Subjects, who agreed to participate in the survey were instructed to click the “Agree to participate” button and then directed them to complete the anonymous self-administered questionnaire.

Ethical clearance was obtained from the Ethical Review Committee of the Macau University of Science and Technology affiliated Zhuhai Science Research Academy (Approval NO. MUST-MEC-20200701XY).

### Measures

The COVID-19 preventive knowledge inventory (including the version of simplified Chinese and traditional Chinese) was adapted from the questionnaire on Chinese residents’ knowledge towards COVID-19 (Table 1) (Panjin Municipal People’s Government, 2020). It had five single-choice questions on heat inactivation conditions for SARS-CoV-2, disinfectants for SARS-CoV-2, transmission ways of SARS-CoV-2, etiquette when coughing and sneezing, and use of disposable masks.

**Table 1:** Questionnaire of COVID-19 preventive knowledge.

		Question list
**Question 1: the time condition and temperature condition that** **can** **inactivate the SARS-CoV-2.**		
	Option A. Maintain 56 °C for 30 min Option B. Maintain 56 °C for 15 min Option C. Maintain 56 °C for 20 min Option D. Maintain below 0 °C for 10 min	
**Question 2: the type of disinfectant that** **cannot** **inactivate the SARS-CoV-2.**		
	Option A. Peroxyacetic acid Option B. 75% ethanol Option C. Iodophor Option D. Bleach powder	
**Question 3: the** **non-transmission** **pathways of the SARS-CoV-2.**		
	Option A. Contact transmission Option B. Droplet transmission Option C. Soil transmission Option D. Aerosol transmission	
**Question 4: the** **wrong** **precaution when coughing and sneezing.**		
	Option A. Cover your nose and mouth with a tissue or elbow when coughing and sneezing. Option B. Cover your nose and mouth with both hands when coughing and sneezing. Option C. Put the sneezing tissue in the dustbin with cover. Option D. It’s best to clean your hands thoroughly after sneezing and coughing.	
**Question 5: the** **wrong** **precaution for the use of disposable medical masks.**		
	Option A. It is recommended to replace the mask every 2–4 h. Option B. It should be replaced immediately once the mask has contaminated. Option C. When wearing masks, avoid touching the inner side of the mask. Option D. The thicker the mask, the better the anti-virus effect.	

The survey questionnaire also collected demographic variables, including gender, age, and type of students (medical *vs.* non-medical).

### Statistical analysis

Correct rates of COVID-19 preventive knowledge were calculated for Macao and Zhuhai students, and compared by using Chi-square test. Because the Macao and Zhuhai samples were not comparable in terms of demographics, multiple logistic regression analyses were used to examine the independent region-knowledge association, which included the answer to each knowledge question (correct/incorrect) as the dependent variable, region (Macao *vs.* Zhuhai) as the main predictor, and demographic variable (including the age, gender and major of students) as covariates. The statistical significance level was set at *p* < 0.05 (two-tailed). Data were analyzed using the Statistical Package for the Social Sciences (SPSS 24.0).

## Results

### Characteristics of the study population

A total of 3,840 participants took part in the survey initially, but 3,795 (98.8%) completed the questionnaire, with 977 from Macao and 2,818 from Zhuhai. As shown in Table 2, in comparison to Zhuhai participants, Macao participants were overrepresented by male students with age younger than 20 years and medical students.

**Table 2:** Characteristics of the respondent.

Variable	*n* (%)		*χ*^*2*^*-*value	*P-*value	
	Macao (*n* = 977)	Zhuhai (*n* = 2,818)			
**Gender**					
Female	530 (54.2%)	1,663 (59.0%)	6.755	0.009	
Male	447 (45.8%)	1155 (41.0%)			
**Age**					
<20	355 (36.3%)	602 (21.4%)	86.247	<0.001	
≥20	622 (63.7%)	2,216 (78.6%)			
**Major**					
Medical	217 (22.2%)	268 (9.5%)	104.984	<0.001	
Non-medical	760 (77.8%)	2,550 (90.5%)			

### Correct rates of COVID-19 preventive knowledge

As displayed in Table 3, Macao and Zhuhai participants had similar correct rates in terms of heat inactivation conditions for SARS-CoV-2 (76.8% *vs.* 76.9%, *P* = 0.950), etiquette when coughing and sneezing (75.9% *vs.* 75.0%, *P* = 0.562), and use of disposable masks (92.2% *vs.* 90.6%, *P* = 0.126), while Macao sample had significantly higher correct rates of disinfectants for SARS-CoV-2 (24.6% *vs.* 17.5%, *P* < 0.001) and transmission ways of SARS-CoV-2 (84.5% *vs.* 79.6%, *P* < 0.001) than Zhuhai sample.

**Table 3:** Correct rates of COVID-19 preventive knowledge in undergraduates of Macao and Zhuhai, China.

Question Subject	*n* (%)		*χ*^*2*^*-*value	*P-*value
	Macao	Zhuhai		
Inactivation conditions for SARS-CoV-2	750 (76.8%)	2,166 (76.9%)	0.004	0.950
Disinfectants for SARS-CoV-2	240 (24.6%)	493 (17.5%)	23.271	<0.001
Transmission ways of SARS-CoV-2	826 (84.5%)	2,244 (79.6%)	11.334	0.001
Etiquette when coughing and sneezing	742 (75.9%)	2,114 (75.0%)	0.336	0.562
Use of disposable masks	901 (92.2%)	2,553 (90.6%)	2.342	0.126

After adjusting for demographic variables (Table 4), the regional differences in correct rates of disinfectants for SARS-CoV-2 (*P* < 0.001) and transmission ways of SARS-CoV-2 (*P* = 0.001) remained statistically significant.

**Table 4:** Logistic regression on the association of correct COVID-19 knowledge with region, adjusted for demographic variables.

Variable^§^	Question Subject																			
	Inactivation Conditions for SARS-CoV-2				Disinfectants for SARS-CoV-2				Transmission Ways of SARS-CoV-2				Etiquette When Coughing and Sneezing				Use of Disposable Masks			
	OR*	95% CI^Δ^		*P*	OR	95% CI		*P*	OR	95% CI		*P*	OR	95% CI		*P*	OR	95% CI		*P*
		Upper	Lower			Upper	Lower			Upper	Lower			Upper	Lower			Upper	Lower	
Region	1.086	0.910	1.297	0.360	0.713	0.595	0.855	<0.001	0.704	0.576	0.861	0.001	0.978	0.821	1.166	0.807	0.809	0.615	1.064	0.130
Age	1.123	0.941	1.342	0.199	1.169	0.975	1.403	0.092	0.855	0.712	1.027	0.094	1.117	0.938	1.329	0.214	1.236	0.944	1.620	0.124
Gender	1.022	0.877	1.191	0.777	1.123	0.951	1.325	0.172	0.754	0.638	0.891	0.001	1.671	1.440	1.939	<0.001	2.123	1.692	2.663	<0.001
Type of students	1.650	1.278	2.131	<0.001	1.957	1.579	2.425	<0.001	1.117	0.872	1.431	0.381	1.391	1.092	1.772	0.008	1.122	0.784	1.606	0.529

**Notes:**

§ Region: 1 = Zhuhai, 2 = Macao; Age: 1 = Less than 20 years old, 2 = 20 years or older; Gender: 1 = Female, 2 = Male; Type of students: 1 = Medical students, 2 = Non-medical students.

* OR: Odds Ratio.

Δ CI: Confidence Interval.

## Discussion

This study compared university students’ knowledge of COVID-19 prevention between Macao and Zhuhai. Students of both cities had more than 75% rate of correct knowledge of heat inactivation conditions for SARS-CoV-2, transmission ways of SARS-CoV-2, etiquette when coughing and sneezing, and use of disposable masks. Although Macao students had statistically significant higher correct rates of disinfectants for SARS-CoV-2 and transmission ways of SARS-CoV-2 than Zhuhai students, which remained statistically significant after demographic variables including age, gender, and types of students were adjusted, the correct rates of disinfectants were still low in students of both cities. Our study results were similar to previous studies (Chang, Yuan & Wang, 2020; Puspitasari et al., 2020).

The higher percentage of medical students in Macao than Zhuhai students cannot fully explain the regional difference in knowledge of disinfectants for SARS-CoV-2 and transmission ways of SARS-CoV-2. The possible reason may be the students in Macao have more hygienic habits, such as wearing masks, good etiquette when coughing and sneezing, and ventilating. Nevertheless, the correct rates of disinfectants for SARS-CoV-2 were low in both cities, because the use of disinfectants requires professional knowledge and lack of students’ knowledge about disinfectants.

The present study has some limitations. First, the two university students were recruited online, so the representation of participants might be limited and need to take caution in generalizing the findings from the current study. Second, the knowledge measures covered in the questionnaire are limited. Items, such as social distancing and hand hygiene, were not included. Finally, although three possible confounding factors were adjusted, the possibility of residual confounding could not be excluded.

Still, findings of the present studies suggest that health education programs are warranted to further improve undergraduates’ knowledge of COVID-19 prevention because approximately 10–15% (Macao SAR) and 25% (Zhuhai) of the students of the two cities were unaware of the transmission ways of SARS-CoV-2 and correct etiquette when coughing and sneezing, respectively. There is also a need to educate students on the correct use of disinfection products during the pandemic. Given that opinion leaders and role models appear to have a key function among students (Abdi & Simbar, 2013), the peer education approach based on social media platforms may be an ideal form of health education among university students. Popular social media platforms, such as WeChat, TikTok, and Weibo, may be a good avenue to spread correct COVID-19 preventive knowledge, similar conclusion drawn in previous study in Pakistani university populations (Salman et al., 2020).

## Supplemental Information

10.7717/peerj.11833/supp-1 Supplemental Information 1 Dataset in Macao.

10.7717/peerj.11833/supp-2 Supplemental Information 2 Dataset in Zhuhai.

10.7717/peerj.11833/supp-3 Supplemental Information 3 Questionnaire for participants.

10.7717/peerj.11833/supp-4 Supplemental Information 4 Questionnaire (in Chinese).

## Additional Information and Declarations

### Data Availability

The following information was supplied regarding data availability: The raw measurements are available in the Supplemental Files.

## References

[ref-1] Abdi F, Simbar M (2013). The peer education approach in adolescents-narrative review article. Iranian Journal of Public Health.

[ref-2] Chang J, Yuan Y, Wang D (2020). Nan fang yi ke da xue xue bao =. Journal of Southern Medical University.

[ref-3] Chinese Center for Disease Control and Prevention (2020). Coronavirus disease 2019. http://www.chinacdc.cn/jkzt/crb/zl/szkb_11803/.

[ref-4] Education and Youth Development Bureau (2020). Staff from the DSEDJ visited the local school to understand the preparation work for the 2020/2021 autumn semester. https://portal.dsedj.gov.mo/webdsejspace/internet/Inter_main_page.jsp?id=77814.

[ref-5] Ministry of Education of the People’s Republic of China (2020). Adhere to the principle of safe, normal, and comprehensive, prevent and control the pandemic scientifically and accurately, and promote the start of the autumn semester steadily and orderly. http://www.moe.gov.cn/fbh/live/2020/52320/sfcl/202008/t20200827_480442.html.

[ref-6] Panjin Municipal People’s Government (2020). Unified examination for prevention and control of COVID-19 (China). http://www.panjin.gov.cn/index.php/survey-post-id-12.

[ref-7] Puspitasari IM, Yusuf L, Sinuraya RK, Abdulah R, Koyama H (2020). Knowledge, attitude, and practice during the COVID-19 pandemic: a review. Journal of Multidisciplinary Healthcare.

[ref-8] Salman M, Mustafa ZU, Asif N, Zaidi HA, Hussain K, Shehzadi N, Khan TM, Saleem Z (2020). Knowledge, attitude and preventive practices related to COVID-19: a cross-sectional study in two Pakistani university populations. Drugs & Therapy Perspectives: For Rational Drug Selection and Use.

[ref-9] World Health Organization (2020). Coronavirus disease (COVID-19). https://www.who.int/emergencies/diseases/novel-coronavirus-2019.

[ref-10] Zhai P, Ding Y, Wu X, Long J, Zhong Y, Li Y (2020). The epidemiology, diagnosis and treatment of COVID-19. International Journal of Antimicrobial Agents.

[ref-11] Zhong B-L, Luo W, Li H-M, Zhang Q-Q, Liu X-G, Li W-T, Li Y (2020). Knowledge, attitudes, and practices towards COVID-19 among Chinese residents during the rapid rise period of the COVID-19 outbreak: a quick online cross-sectional survey. International Journal of Biological Sciences.

