# Supplementary material for: A comparative study on knowledge towards COVID-19 prevention among undergraduate students in Macao and Zhuhai, China
Source: PeerJ. 2021 Aug 3;9:e11833. doi: 10.7717/peerj.11833 (PMC8344680; doi:10.7717/peerj.11833)
Supplement: Supplemental Information 3 [file peerj-09-11833-s003.pdf]

Questionnaire of COVID-19 preventive knowledge

Question list
**Question 1: The time condition and temperature condition that can inactivate the SARS-CoV-2.**
Option A. Maintain 56 ℃ for 30 minutes
Option B. Maintain 56 ℃ for 15 minutes
Option C. Maintain 56 ℃ for 20 minutes
Option D. Maintain below 0 ℃ for 10 minutes
**Question 2: The type of disinfectant that cannot inactivate the SARS-CoV-2.**
Option A. Peroxyacetic acid
Option B. 75% ethanol
Option C. Iodophor
Option D. Bleach powder
**Question 3: The non-transmission pathways of the SARS-CoV-2.**
Option A. Contact transmission
Option B. Droplet transmission
Option C. Soil transmission
Option D. Aerosol transmission
**Question 4: The wrong precaution when coughing and sneezing.**
Option A. Cover your nose and mouth with a tissue or elbow when coughing and sneezing.
Option B. Cover your nose and mouth with both hands when coughing and sneezing.
Option C. Put the sneezing tissue in the dustbin with cover.
Option D. It's best to clean your hands thoroughly after sneezing and coughing.
**Question 5: The wrong precaution for the use of disposable medical masks.**
Option A. It is recommended to replace the mask every 2-4 hours.
Option B. It should be replaced immediately once the mask has contaminated.
Option C. When wearing masks, avoid touching the inner side of the mask.
Option D. The thicker the mask, the better the anti-virus effect.
