# Supplementary material for: A comparative study on knowledge towards COVID-19 prevention among undergraduate students in Macao and Zhuhai, China
Source: PeerJ. 2021 Aug 3;9:e11833. doi: 10.7717/peerj.11833 (PMC8344680; doi:10.7717/peerj.11833)
Supplement: Supplemental Information 4 [file peerj-09-11833-s004.pdf]

问卷编号：□□-□□□□□□□□□　　　问卷录入 1：________________

问卷审核：________________　　　问卷录入 2：________________

大学生新冠肺炎疫情预防知识知晓率调查问卷

（简体字版）

同学，你好！

2020 年初，在毫无征兆的情况下，新冠疫情席卷全球，给人们的健康乃至社会经济发展带来了严重的威胁。确保学生能够在此类公共卫生突发事件爆发后仍能保持正确的预防行为至关重要。为了评估大学生对新冠肺炎预防的知识储备，我们邀请你参与本次匿名在线调查。完成本次调查大约需要 **5** 分钟左右，问卷内容不涉及个人隐私信息，你的所有填答信息将做保密处理，请你按照你的情况如实填答问卷，十分感谢你的支持和参与，谢谢！

澳门科技大学、吉林大学珠海学院联合课题组

2020 年 **8** 月

第一部分 知情同意授权

在填答开始前需要你先点勾选下方对应选项，谢谢配合！

○同意参加　○不同意参加

第二部分 基本资料

1.你的出生年月：_______年_______月

2.你的性别：○女　○男

第三部分 疫情相关知识

1.针对新型冠状病毒，你认为以下哪种条件**可以**杀灭病毒：

○在 56 ℃，30 分钟条件下　○在 56 ℃，15 分钟条件下

○在 56 ℃，20 分钟条件下　○在＜0 ℃，10 分钟条件下

2.你认为下列哪种消毒剂**不能**有效灭活新冠病毒：

○过氧乙酸　○75%乙醇　○碘伏　○漂白粉

3.你认为新冠病毒的传播途径**不包括**一下哪一种：

○接触传播　○飞沫传播　○土壤传播　○气溶胶传播

4.关于咳嗽和打喷嚏时的注意事项，你认为以下说法**不正确**的是：

○打喷嚏时应用纸巾或胳膊肘遮掩口鼻　○咳嗽和打喷嚏时应用双手遮掩口鼻

○把打喷嚏用过的纸巾放入有盖垃圾桶　○打喷嚏和咳嗽后最好彻底清洁双手

5.关于口罩的使用事项，你认为以下说法**不正确**的是：

○建议每 2-4 小时更换一次　○一旦污染，应第一时间更换

○佩戴时，避免手接触口罩内侧面　○口罩越厚，防病毒效果越好

問卷編號：□□-□□□□□□□□□　　問卷錄入 1：________________

問卷審核：________________　　問卷錄入 2：________________

大學生新冠肺炎疫情預防知識知曉率調查問卷

（繁體字版）

同學，你好！

2020 年初，在毫無征兆的情況下，新冠疫情席卷全球，給人們的健康乃至社會經濟發展帶來了嚴重的威脅。確保學生能夠在此類公共衛生突發事件爆發後仍能保持正確的預防行為至關重要。為了評估大學生對新冠肺炎預防的知識儲備，我們邀請你參與本次匿名在線調查。完成本次調查大約需要 **5** 分鐘左右，問卷內容不涉及個人隱私信息，你的所有填答信息將做保密處理，請你按照你的情況如實填答問卷，十分感謝你的支持和參與，謝謝！

澳门科技大学、吉林大学珠海学院联合课题组

2020 年 **8** 月

第一部分 知情同意授權

在填答開始前需要你先點勾選下方對應選項，謝謝配合！

○同意參加　○不同意參加

第二部分 基本資料

1.你的出生年月：_______年_______月

2.你的性別：○女 ○男

第三部分 疫情相關知識

1.針對新型冠狀病毒，你認爲以下哪種條件<u>可以</u>殺滅病毒：

○在 56 ℃，30 分鍾條件下　○在 56 ℃，15 分鍾條件下

○在 56 ℃，20 分鍾條件下　○在＜0 ℃，10 分鍾條件下

2.你認爲下列哪種消毒劑<u>不能</u>有效滅活新冠病毒：

○過氧乙酸　○75%乙醇　○碘伏　○漂白粉

3.你認爲新冠病毒的傳播途徑<u>不包括</u>壹下哪一種：

○接觸傳播　○飛沫傳播　○土壤傳播　○氣溶膠傳播

4.關于咳嗽和打噴嚏時的注意事項，你認爲以下說法<u>不正確</u>的是：

○打噴嚏時應用紙巾或胳膊肘遮掩口鼻　○咳嗽和打噴嚏時應用雙手遮掩口鼻

○把打噴嚏用過的紙巾放入有蓋垃圾桶　○打噴嚏和咳嗽後最好徹底清潔雙手

5.關于口罩的使用事項，你認爲以下說法<u>不正確</u>的是：

○建議每 2-4 小時更換一次　○一旦汙染，應第一時間更換

○佩戴時，避免手接觸口罩內側面　○口罩越厚，防病毒效果越好
